# Epidemiologic Trend of Smear-Positive, Smear-Negative, Extra Pulmonary and Relapse of Tuberculosis in Iran (2001-2015); A Repeated CrossSectional Study

**Published:** 2017-05-20

**Authors:** Shahram Arsang-Jang, Marjan Mansourian, Firouz Amani, Tohid Jafari-Koshki

**Affiliations:** ^1^ Department of Biostatistics and Epidemiology, School of Health, Qom University of Medical Sciences, Qom, Iran; ^2^ Department of Biostatistics and Epidemiology, School of Health, Isfahan University of Medical Sciences, Isfahan, Iran; ^3^ Department of Biostatistics and Epidemiology, School of Medicine, Ardabil University of Medical Sciences, Ardabil, Iran; ^4^ Department of Biostatistics and Epidemiology, School of Health, Kermanshah University of Medical Sciences, Kermanshah, Iran

**Keywords:** Iran, Poisson distribution, Relapse, Tuberculosis

## Abstract

**Background:** Trend analysis is an important tool to monitor epidemiological changes of disease over time to guide resource allocation. This study aimed to study incidence trends and change-points of smear positive, smear negative, extra-pulmonary and relapse of tuberculosis (TB) in Iran from 2001 to 2015.

**Study design:** Repeated cross-sectional study

**Methods:**Nonlinear segmented regression w as used to describe TB incidence trends; annual percent change (APC), average annual percent changes (AAPC) and change points for each disease separately.

**Results:** Of 154930 TB cases, 49.8% w ere smear positive, 19.7% smear negative, 27.32% extrapulmonary and 3.18% relapse. For all TB types, the peak of incidence w as in 2001. Tw o change point w ere estimated for all TB types (P<0.05). The APC of all TB types w ere -6.51 (95% CI: -7.4, -5.4) for first and 2.4 (95% CI: 0. 7, 4.1) for second segment. Although the trends w ere significantly decreasing from 2001 to 2015 for smear positive (AAPC=2.06%), smear negative (AAPC=3.57%), extra pulmonary (AAPC=3.2%) and relapse (AAPC=3.3%), the AAPCs of trends w ere not significant from 2006 to 2015. Except for Extra pulmonary TB (APC=4-.9%, 95%CI:-10, 1.2), the APCs of the last segments w ere significant.

**Conclusions:**Even though the TB incidence rates w ere decreasing, the amount of reductions seem inadequate, to reach the goals of TB control in Iran. Especially, the increase in the extra-pulmonary TB rates is a point of concern that highlights more attention is required for these cases. It is essential to improve economic supports tow ard TB control, illegal immigrants, data registry systems and physician's sensitivity in TB detection.

## Introduction

Tuberculosis (TB) causes ill-health in millions of people each year and it was one of the top 10 causes of death worldwide in 2015^1^. According to last report WHO in 2015, there were an estimated 10.4 million new TB cases worldwide (including 56% among man and 10% among children)^1^. In addition, the 6314151 new cases notified that 14.5% and 6.5% of them were Extra-pulmonary TB (EPTB) and relapse cases, respectively^2^. Additionally, one of the five detected cases of TB has EPTB form^3^. There were also 1.8 million death from TB cases including HIV positive (0.4 million), and death in men was 2.48 times approximately higher than in women (62%/25% deaths) ^1^.

As an intermediate TB burden country, 10290 new TB cases were reported in Iran in 2015, of which 4924 (48.61%) were smear positive (SP), 2863 (28.26%) EPTB, 1478 (14.59%) smear negative (SN) and 299 (2.95%) relapse cases^4^. The highest relative risk of SP incidence rate was related to provinces of Sistan-Baluchestan (relative risk=4.2) followed by Golestan (relative risk =3.18) and Khorasan-Razavi (relative risk =1.67)^5, 6^.

For control of TB, implementation of treatment short-course and BCG vaccination has been started in Iran from 1984. Subsequently, the treatment short-course was replaced with DOTS strategy in 1995^4^. In spite of this, like many other countries, TB remains major cause of health problems in Iran^7^. The target for TB control strategy is to have <1 case per 1 million per year until 2050 and an 80% decline in the TB incidence rate by 2030, compared with 2015^1^. Study the trend of incidence or mortality rates over time provides important measures for assess the effect of controlling programs and health policies for government, and understanding the mechanisms behind observed changes to develop corresponding control strategies^8, 9^.

Various models are used for determining the trend of incidence or mortality rates. Segmented regression models is popular models of trend analysis in incidence or mortality rate to estimate the changes rate and changes in time. In this model, the trend can be described by Annual Percent Change (APC) and Average Annul Percent Changes (AAPC) ^10, 11^.

APC and AAPC provide summery statistics that facilitate visual comparisons of disease incidence. Studies are used segmented regression models to analysis the trend of TB incidence rate. In this model, APC is estimated by fitting a simple linear model on logarithm of rates regressed on time. Constant rate of change for each segment is assumption of implementation of APC. AAPC describes and compares the rate of change that has not limitation of APC and will be same when there is no transition on trend^8^. There was no study to evaluate national TB trend base on TB types in Iran. Moreover, the most studies were conducted based on local information^12^.

The objective of this study was to assess the efficacy of TB controlling programs in Iran by determining the extent changes of SP, SN, EPTB and relapse incidence rate per 100000 population during 2001-2015 and to estimate point in time point at which the TB changed.

## Methods

In this repeated cross-sectional study, we provided the incidence rates on notified smear-positive, smear-negative, extra-pulmonary and relapse TB cases from 21 March 2001 to 21 March 2016, following WHO guidelines, coming from the reports published by the Ministry of Health of Iran, which hosts the national TB surveillance system. We excluded cases were HIVpositive, because of unavailable information. The study area consisted of 31 provinces of Iran with total landmass of 1,648,195 km^2^ and a population of over 80 million inhabitants (2016 population census). The Alborz Province before 2010 was part of Tehran Province, so data of these provinces were analyzed together (merged).

In Iran, TB controlling programs are included in healthcare systems since 1990, further implementation of DOTS strategy and "DOTS II" strategy started since 1996 and 2010, respectively. The TB surveillance programmer uses passive case finding. According to the WHO recommendation, TB patients are treated with an intensive phase of 2 months, followed by a *continuation phase* of 4 months regimens. Currently, there are 497 free TB diagnostic and treatment centers in Iran with at least one cultivation center in each province. These centers quarterly report data from verified TB cases to the “Administration of Tuberculosis and Leprosy Control” of Ministry of Health and Medical Education using electronic forms. In addition, annual reports on findings for TB control activities are provided for national use.

In this longitudinal analysis, we imported the logarithm scale of TB incidence crude rates as dependent variable and year as independent variable to model. A crude incidence rate is the number of new TB cases in a specified population during a year $\left(\frac{new\;cases}{Population}*10^{5}\right)$ . In situation that data arise from Poisson distribution, which is skewed log, transformation makes it approximate a normal distribution. Selection the maximum number of change-points in model depends on number of years (n), so, to identify change in trends, with regarding n= 15 years, segmented regression model with maximum 4 number of change-points was used^10^. Segmented linear regression is used for segmenting nonlinear regression models into linear segments and points between segments called change points^11^.

The segmented regression has the following specification:

E(y|x)=β_i,0_+β_i,1_x+δ_i,1_(x-τ_i,1_)^+^+…+δ_ i,k_i__(x-τ_ i,k_i__)^+^

Where k_i_ is unknown number of change points for *ith* group (i=1,2), τ_i,l_ (i=1,.., k) is the unknown change points, β_i,l_ and δ_i_ are the regression parameters and (x-τ_i,j_)^+^=(x-τ_i,j_) for(x-τ_i,j_)>0 . The details of segmented regression and APCs discussed previously.

The Lerman’s grid search was used on τi,j, that uses last square estimation method, which carries out a grid search over trend to map minimum value of residuals sum of square function^13^. The APC, AAPC and its respective 95% confidence interval (95%CI) were used for description trend of TB. The heteroscedastic error was used to model the trends as the Poisson model. The comparability test was used to test whether two regression mean functions were parallel. The *P*-value was estimated using permutation test of Monte Carlo.

We used Bayesian Information Criterion (BIC) to choice final Model. Model with smallest BIC was considered as best model. Free source Join point 4.4.0 software available from http://srab.cancer.gov/joinpoint/ was used to perform this analysis.

Due to retrospective nature of the study ethical approval was not required.

## Results

**Figure 1:**
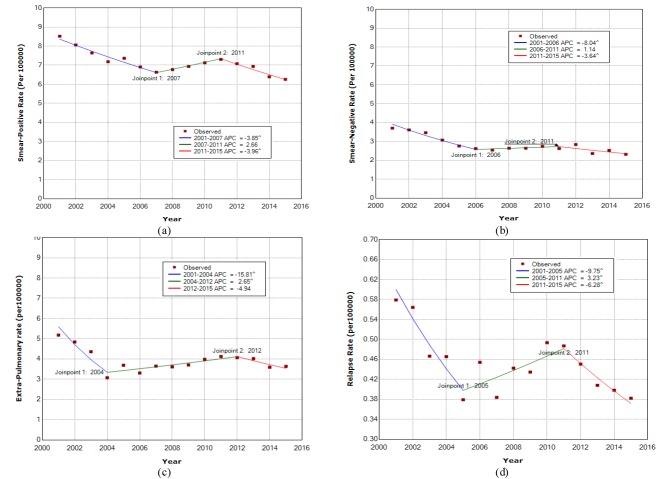
Incidence rate trend of Smear-positive (a), Smear-Negative (b), Extra-pulmonary(c) and relapse rate (d) of TB in Iran during 2001-2015. The annual percent change (APC) is significantly different from zero at alpha=0.05

Between 2001- 2015, a total of 154930 TB cases were registered by health surveillance systems in Iran. Of these, 77155 (49.8%) were SP, 42327 (27.3%) were EPTB, 30521 (19.7%) were SN and 4927 (3.1%) were relapse cases. For all types TB, the peak of incidence was related to 2001. Besides, the full range AAPC related to all forms of TB was -2.6 (95%CI: -3.3, -1.9, *P*≤.0001) with two change-points in 2006 and 2011. In addition, the trend of first (APC=-6.51, 95%CI: -7.4, -5.4) and third (APC=-3.7, 95%CI:-5.4, -2.1) segments were downward, however, it was upward for second segment (APC=2.6, 95%CI: 1.3, 4). Figure 1a-d show the estimated trend of TB incidence rate for each form over 15 years. Accordingly, using the join point regression analysis, two change-points on incidence rate trend for all four TB forms were estimated. The pattern of trends for all TB forms were nearly same despite of different change times and rates.

The AAPC results of SP, SN, EPTB and relapse incidence rates of TB for the 15 years, from 2006 to 2015 and from 2011 to 2015 are shown in Table 1.

**Table 1:** Average annual percent change (AAPC) results of tuberculosis in Iran during 2001-2015

**Period**	**AAPC**	**95% CI for AAPC**		**Test sttistic**	***P*** ** value**
Smear Positive				
2001-2015	-2.06	-3.05	-1.07	-4.05	0.001
2006-2015	-1.05	-2.49	0.398	-1.42	0.153
2011-2015	-3.95	-5.90	-1.90	-4.64	0.002
Smear Negative				
2001-2015	-3.57	-5.02	-2.07	-5.08	0.001
2006-2015	-1.01	-3.08	1.06	-1.03	0.300
2011-2015	-3.64	-7.10	-0.071	-2.57	0.037
Extrapulmonary				
2001-2015	-3.2	-4.85	-1.55	-3.75	0.001
2006-2015	0.05	-1.82	1.96	0.05	0.954
2011-2015	-3.09	-6.84	0.81	-1.55	0.119
Relapse					
2001-2015	-3.36	-5.10	-1.58	-3.67	0.001
2006-2015	-1.10	-3.18	1.01	-1.03	0.303
2011-2015	-6.27	-10.43	-1.92	-3.37	0.012

Therefore, for all forms of TB, AAPCs of incidence rate trend for 15 years were significantly different from zero, the AAPCs from 2006 to 2015 and from 2011 to 2015 were significant at alpha=0.05. The highest reduction in incidence rate was estimated for SN TB at 3.6%, and the lowest reduction in incidence rate for SP TB at 2.1%.

The estimated expected mean of segmented regression function for TB incidence rate were:

E(y|x)=80.65-0.039x+0.065(x-2007)^+^-0.066(x-2011)^+^ (Smear positive)

E(y|x)=168.9-0.083x+0.095(x-2006)^+^-0.048(x-2011)^+^ (Smear Negative)

E(y|x)=345.98-0.172x+0.198(x-2004)^+^-0.07(x-2012)^+^ (Extra-pulmonary)

E(y|x)=204.74-0.102+0.13(x-2005)^+^-0.096(x-2011)^+^ (Relapse)

The segmented regression function demonstrates the expected SP incidence rate equal to 2.51 in 2050. Regarding the last segment without any change in trend, we can expect that the SP incidence rate to be 1.66 per 100000 in x =2050.

Hypothesis of parallelism trends between SP TB (*P*=0.460), SN TB (*P*=0.090) and EPTB trend (*P*=0.673) with relapse trend were accepted. The parallelism hypothesis of trends for all forms with refugee trend (*P*<0.001) as well as Gross Domestic Product (GPD) trend (*P*<0.0001) were rejected.

## Discussion

This study was strikingly first national TB trend analysis after implementation DOTS strategy in Iran. The trend of TB in Iran has changed over the past 15 years. The estimated decreasing trend for first and third segments also increasing trend for second segment for all form TB incidence rate are generally in line with previous studies in Iran^14^. As a results of AAPC_15years_ decreasing was slower than previous study in Iran,^15, 16^ where both AAPC_10year_ (1998-2008) and AAPC_5year_ (2003-2008) were 4.5%. Moreover, it was slower than those of high-income countries were (annually 3.6% decries) ^17^. However, decreasing rate is steeper than both globally (AAPC_10year_=-1) and Eastern Mediterranean region (AAPC_10years_= -0.08) during 2001-2010^18^. Considering last segment pattern for trend; hence, we expect about 52% reduction in TB incidence rate by 2030, compared with 2015. This demonstrates need for accelerating reduction rate to achieve the first milestones of the End TB Strategy (80% reduction) in Iran, attributable mainly to the second segment, and highlights the necessity to investigate the related factors. Subgroup analysis in different TB types could shed light on unknown aspects of the trend.

The SP, SN, EPTB and relapse TB incidence rate declined significantly from 2001 to 2015, however, the trend of EPTB incidence rate was not significant from 2011 to 2015. The exact information underlying this pattern is unknown, but various factors such as poor access to primary health care centers, diagnostic delays, ethnicity, education, economic condition, immigration and pollution are involved in TB trend^19^. However, in this study the effects of immigration and GPD trend on national TB trend were not significant. In Iran, immigrants are allowed to settle only in some predetermined provinces, so it will be important to analyze parallelism of trend in province levels. As a report of ministry of Iran, from 2005 to 2010 about 5000000 Afghani with illegal migration returned to Afghanistan. This population has an important role in TB trend, because of deportation fear; most of them do not refer to health care centers. Moreover the illegal migrations are not considered in reported immigration trend. In addition, economic level as well as health budget distributions are not equal among provinces in Iran^20^. These interactions may reduce the effects of immigration and economic factors on TB trend.

The estimated changes point in TB trend were similar for all forms, therefore it can be due to health care systems activities in Iran. The inadequate number of laboratories for TB, in addition, delay in TB detection can cause fix trend for second segments. There were upward trend for TB detection (AAPC=2.5) and fix trend for successful treatment between 2001-2008 in Iran.^16^ Moreover, delay in TB detection is an important problem, and the mean of delay varied between 100 and 216 days for different type of TB during 2006-2012 in Iran^21^. In addition, bias in EPTB and SN cases diagnosis is common when there is no adequate laboratory for confirmatory tests^22^. About 67.5% of patient with TB, in first time contact with private sectors^23^, in addition, only 17% and 7.5% of private sectors knew about TB diagnostic and treatment methods^24^. As a results of nonlinear trend for TB, it seems that effect of controlling programs were varied over 15 years, hence for success of TB control it will be important to; 1) Shorten inclusion of periodic courses for physicians in TB controlling programs, 2) Increase knowledge and sensibility of illegal immigrants related to TB and 3) Increase the number of local laboratory.

Results of this model for SP trend is the same with spatiotemporal model^6^, where estimated national trend was declined at a rate of 3.7% for 10 years_ (2001-2012)_ in Iran. Downward trend for last segment of SP TB may be due to improved health system strengthening following economical support from the Global Fund to Fight AIDS, tuberculosis and malaria for proposed TB control programs in 2008. After that, the local and regional laboratories for drug susceptibility testing are increased from 0.1 to 0.4 per 5 million population in 2011 as well as TB diagnostic service using culture from 2.6 to 3.2 per 5 million population in 2010. In addition, MDR treatment centers were prepared, that have important effect on early detection rate. However, economic level was related with TB incidence rate trend^18^, but in this study, there was no parallelism trend between GDP and SP TB. It seems that improvement in economic level must be in direction that enhance facilities related to TB controlling programs. Regarding that there was no change in TB diagnostic methods and registration in this period, therefore, it is important to improve TB diagnostic and therapeutic facilities over time for reach the elimination of TB. In addition, regarding the previous studies in Iran it may result in treatment problem, where, during 2001-2008, case detection rate and success treatment rate were upward and fixed, respectively^16^. However, there is no uniform pattern for trend in provinces level, so we suggest estimating TB trend in provinces level for assessing health service programs.

The SN TB incidence trend was relatively downward from 2001 to 2006 and it was stable during 2006-2011, which is lower than the raising trend of 4.5% from 2001 to 2009 in Iran^16^. From 2005 to 2011, SN TB decreased with 30% annual rate in province level (Hamadan) of Iran^25^. It was downward in Saudis and non-Saudis during 2000 to 2009^26^. The stable trend of SN TB may result in lower respiratory symptoms in these patients in comparison with SP TB patients, also diagnostic procedures of SN TB contrast SP TB are complicated, which may cause to different change point on trend result in delayed detection^27^. About half of SN cases will be transformed to SP TB without treatment^28^, hence, same pattern of trends between SN and SP is unavoidable. Hoverer, the trend of SN TB with SP TB was not parallel, which can result in different change-points on trends.

About 27% (range: 20.5-29.2%) of TB cases were EPTB with stable trend of ratio during the study period. An EPTB rate with a range of 13- 48% was reported in Iran^29^. EPTB proportion of all TB cases increased from 48% to 53% from 2000 to 2006 and 15.7% to 21% from 1993 to 2006 in England and US, respectively^30, 31^. But this was higher than 22% reported for EMRO regions in 2014^2^, possibly because of variation in population and diagnostic methods. Our study shows that the trend of EPTB incidence rate remained stable from 2011 to 2015 and from 2006 to 2015, but since the trend of pulmonary TB was downward, this has caused to an increase in the EPTB ratio among all TB cases in Iran. However, this increases was not significant. The EPTB is often perceived more as a clinical property than a public health problem, as unpublished results of our work shows that case detection rate was stable during 2006-2011 and then decreased significantly since 2011 in Iran, so the upward trend of second segment and stable trend of EPTB for third segment maybe related to increasing incidence of EPTB cases in Iran, and probably is not a result of over-diagnosis.

We observed decreasing trend in relapse rate between 2001 and 2015, although fluctuations were seen. In this study relapse incidence rate varied between 0.051 – 0.07 per 100000 population, that it is higher than the reported relapse incidence rate in Golstan (0.034 per 100000), as a high TB burden provinces in Iran, in 2009^7^. The average relapse rate was estimated as 9.62% from 2006 to 2010 in Korea^32^. Accordingly, it was higher than the 0.04 probability of recurrent TB in ideal conditions^33^ and 4.3% relapse proportion in Taiwan during 2004-2008, this ratio can lade to same pattern change of relapse and TB incidence. The slope of decreasing for last segment was slightly steeper than other TB forms. During the period from 2011 to 2014, three MDR TB inpatient centers and eight regional laboratories began to provide TB control services in Iran. Additionally, national online TB guide examination was conducted from 2012^4^. Considering second change-points, these improvements have important role in decreasing TB incidence rate. SP, SN and EPTB are associated with a higher risk of relapse^34^. Therefore, efforts to control and management all other type TB can be important to decreasing relapse cases. Classification of patients for treatment of TB into high risk and low risk groups can be effective in reduction of relapse cases^34^.

There are several limitations to our study, first, incomplete data from individual as well as variables on demographic and behavioral factors, and socio-economic factors were not collected. However, in new registry systems this factors is included, this make difficult explanation of trends. Second, studies of TB trend in country level is too low, so this makes it difficult to compare the results. The segmented regression is inadequate to regress data including outliers, so it is necessary to extension this model to overcome the outlier problems.

## Conclusions

All TB forms have decreased from 2001 to 2015 with different rate, while it remains as a health problem in Iran and is important to accelerating the reduction rate. The increase in the EPTB rates with respect to the other forms of TB cases is a point of concern highlighting the importance of strengthening the services towards this cases. There are association among expanding laboratories and private TB treatment hospitals with change-points on TB trend. Although association among TB trend with immigration and GDP were insignificant, attention must be paid to illegal immigration and financial support toward TB controlling programs. For success TB control, specifically relapse cases because of high variability, will be important to inclusion continues programs along with short- interval monitoring based on provinces and high risk groups. This study would help better understanding of the trend pattern of TB and provide important information for the improve stop TB strategies. Cluster provinces based on TB trend risks for assign resources according to severity of TB controlling problems is recommended.

## Acknowledgements

We gratefully acknowledge the staff from Ministry of Health of Iran who have collected and reported data about TB cases.

## Conflict of interest statement

None.

## Funding

This study supported by Qom University of Medical Sciences. The information reported in this paper is independent from the funding sources.

## Highlights

- Reduction on TB incidence rate have decreased from 4.5% to 3.3% per year.
- Upward and fix trend of Extra pulmonary incidence rate trend is a point of concern.
- There were association among expanding the number laboratories and private TB treatment hospitals with change-points on TB trend.
- There were non-parallelism trend among TB incidence rate and immigration as well as GPD
